# An ecosystems approach to mental health services research

**DOI:** 10.1192/bji.2020.24

**Published:** 2021-02

**Authors:** Mary Anne Furst, Nasser Bagheri, Luis Salvador-Carulla

**Affiliations:** Australian National University College of Health and Medicine. Email: Mary.Furst@anu.edu.au

**Keywords:** Mental health ecosystems, mental health services research, mental healthcare delivery

## Abstract

Mental health ecosystems research is an emerging discipline which takes a whole-systems approach to mental healthcare, facilitating analysis of the complex environment and context of mental health systems, and translation of this knowledge into policy and practice. Evidence from the local context is needed in the analysis of complex interventions and of geographic variations in the outcomes of care. Technical tools and support have been developed to gather and interpret evidence from the local context and translate it in a meaningful and relevant manner for planners and policy makers to guide their decision-making.

Health ecosystems refer to the totality of the circumstances that relate to a given health phenomenon in a defined environment. They comprise the elements which together provide capital to ‘sustain and enhance human wellbeing’, including natural capital such as green spaces, and social capital, which includes both built (infrastructure) and human (institutions and human governance) capital.^1,2^ A population health system includes four main domains: the places and communities in which we live; the wider determinants of health (for example, the social and demographic characteristics of the environment); our health behaviours and lifestyles; and integrated healthcare provision^3^ at the different levels of the ecosystem (nano (patient–professional level), micro (service level), meso (local area level) and macro (region/country level)). The mental health ecosystem is a subset of the general health system which focuses on domains relevant to mental health, such as the characteristics of the population at risk of or suffering mental illness, the workforce and organisations providing care and support to this target population, and their connections, for example, clinician–patient contacts, and the relationships between patients and organisations and among organisations.

Mental health ecosystems research is a part of implementation sciences, which facilitates analysis of environment and context, and knowledge translation to policy and practice. It incorporates an array of different disciplines, including systems dynamics, context analysis, health economics and knowledge discovery from data. It moves away from a reductionist approach focused on developing individual solutions to complex problems, towards providing an analysis of the environment and context of mental health systems and the development of decision support tools to guide policy makers. This analysis of the context of mental health systems – i.e. of local conditions and system behaviour – can help policy makers and researchers to understand geographic variation in care delivery outcomes, where an intervention which has been implemented successfully in one location has produced a different outcome in another. As shown by international studies of assertive community treatment, the effect of an intervention depends on characteristics of the local context. This indicates that full fidelity to the original model in local implementation of a complex intervention may be questionable unless the local context is considered.^4^

Evidence from the local context of care can support decisions about relevant issues such as effectiveness, equity and access to healthcare provision. It requires an approach that goes beyond the traditional evidence-based model^5^ and should incorporate a broader concept of scientific knowledge in systems research, with methods and tools developed in other areas of systems research such as policy decision-making in environmental sciences. This broad approach to scientific knowledge, which incorporates experimental, observational and local evidence together with expert and experiential knowledge for health systems research, has been described in detail elsewhere.^6,7^

Ecological science and the study of biological ecosystems and the services they provide to humans (ecosystem services (ESS)) have provided a conceptual framework on which mental health ecosystems can draw: the Intergovernmental Science-Policy Platform on Biodiversity and Ecosystem Services (IPBES).^2^ Like mental health systems research, ESS research brings together knowledge of a broad and often highly complex social, economic and institutional context from researchers in a range of domains and disciplines, with different levels of expertise and experience, and from different research methods. It also includes relevant knowledge held by non-scientific experts on aspects of the local context, for example, indigenous or local culture (in the case of ESS) or implicit knowledge of the workforce (in the case of mental health services). The ESS framework includes the types of capital (natural, built and social) which together improve human well-being, to which we can add mental capital (the mental health service system). Figure 1 shows adaptations of the IPBES approach to scenarios and modelling to policy decision-making in mental health.

**Fig. 1:**
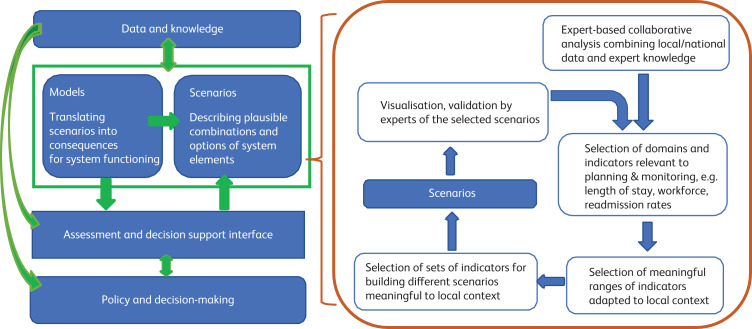
Modified IPBES conceptual framework (summary for policymakers of the methodological assessment of scenarios and models of systems of mental health care delivery).

Practical tools for mental healthcare ecosystems research include: (a) logic models and conceptual maps of the system, and taxonomies of critical domains and characteristics, for example, classifications of mental health services, lifestyles, demographic characteristics or health system indicators; (b) visual tools including geographical information systems; (c) composite or synthetic indices; (d) integrated atlases and maps of the service delivery system, the financing flows or the spatial epidemiology of the target condition; (e) navigation tools for consumers and professionals; (f) decision support systems (DSS) that incorporate the former tools, and artificial intelligence, machine learning and other techniques for knowledge discovery from databases; and (g) impact analysis tools to monitor the adoption and performance of the DSS.

In addition, the modified mental health matrix framework^8^ allows us to identify all these components, domains and indicators and apply tools at different levels of the ecosystem (macro or country/regional level (level 1), meso or local area level (level 2), micro or service level (level 3), and nano or individual consumer level (level 4)); and at different stages in the process of care (A = input, B = throughput, C = output) (Table 1). Examples of this approach to guiding policy have been developed for regional planning in Catalonia and the Basque country in Spain, and in Finland and Chile, using the ESMS/DESDE (European Service Mapping Schedule/Description and Evaluation of Services and Directories).^9^ For example, the agency for mental health planning in Catalonia has constructed a series of integrated atlases of mental healthcare that include health, social, education, employment, justice and housing services.^10^ These atlases have been used to monitored the evolution of the system from 2002 to 2017, identifying system changes before and after the implementation of the 2006 regional mental health plan, and the effects of the global financial crisis in this region from 2008 to 2015. This information has been used to carry out spatial analyses of the prevalence of mental disorders and related sociodemographic factors, both in the whole region and in metropolitan areas. It has been used with analysis of service utilisation, burden and costs of mental illness to feed models of comparative technical efficiency and self-organising mapping networks within the region and in comparison with other regions in Spain.^11^ This holistic approach facilitates the analysis of health improvement under conditions of uncertainty and broadening of the patterns of service provision as suggested by the meta-community model of mental healthcare.^12^ The meta-community model describes a suite of aims including coordinated systems providing care for people with mental illness at a comparable level to that provided for people with physical illness, delivered flexibly and innovatively to people in a range of settings in addition to healthcare settings, such as prisons, asylums, schools and refugee settings.

**Table 1:** Modified mental health matrix^8^

	Input (A)	Throughput (B)	Output (C)
**Macro Country/region (1)**	1A	1B	1C
**Meso Local area (2)**	2A^a^	2B	2C
**Micro Service (3)**	3A	3B	3C
**Nano Individual (4)**	4A	4B	4C

a. This is the level of analysis of the ESME/DESDE approach: input to the system at service level.

An ecosystem approach to health systems research is particularly relevant in the study of the characteristics and dynamics of complex mental healthcare systems. This approach recognises the limitations of traditional research methods when dealing with situations of complexity. It is informed by research in other areas, including ecological science. Progress has been made in the development of technical supports and instruments using an ecosystems approach and collaborating with local domain expertise to ensure relevance and meaning for decision makers and so for the development of evidence-informed policy.

## Supplementary material

For supplementary material accompanying this paper visit https://doi.org/10.1192/bji.2020.24. click here to view supplementary material
